# Medical specialist distributions in Ecuador: a geographical and temporal analysis of data from 2000 to 2017

**DOI:** 10.1186/s12913-022-08056-5

**Published:** 2022-05-19

**Authors:** Alejandro Rodriguez, Adriana Romero-Sandoval, Bernardo A. Sandoval, Natalia Romero

**Affiliations:** 1 Escuela de Medicina, Universidad Internacional del Ecuador, Quito, Ecuador; 2 Escuela de Relaciones Internacionales, Universidad Internacional del Ecuador, Quito, Ecuador; 3 Red Grups d’Amèrica i Àfrica Llatines – GRAAL, Barcelona, Spain

**Keywords:** Medical specialties, Health workforce, Ecuador, Physicians

## Abstract

**Background:**

Knowledge of medical specialists' numbers and geographical distribution are essential for planning health services and health workforce supply. However, although the distribution of physicians is a significant concern for society and policymakers in Ecuador, no studies have evaluated the distribution of specialists in the country. This study aimed to explore the geographical and temporal distribution of medical specialists in Ecuador over 18 years from 2000 to 2017 and analyse its implications for health planning and medical training.

**Methods:**

We conducted an ecological time-series study based on the National Statistical Register of Resources and Health Activities data. This register provides administrative information for health professionals working in public and private health institutions. Rates of medical specialists by year, geographical area, and speciality were estimated. We used joint-point analyses to identify time trends for medical specialists and physicians in training.

**Results:**

From 2000 to 2017, medical specialists grew from 2737 to 10,929. The rate of medical specialists per 10,000 population increased from 4 in 2000 to 10.3 in 2017. Based on Joint point analysis, two temporal trends were identified. Between 2000 to 2015, specialists increased by 4.1% per year, and between 2015 and 2017, they increased by 20% per year. For the entire study period, three cities (Quito, Guayaquil, and Cuenca) accounted for more than 50% of the specialists in the country. However, medical specialists in other cities and rural areas increased from 37% in 2000 to 46% in 2017. The provinces of Esmeraldas, Carchi, Bolívar and Los Ríos presented rates of less than 6 specialists per 10,000 population by 2017. Of the 46 medical specialities identified by 2017, three represented more than 30% of the professionals (gynaecology 12%, paediatrics 11% and family and community health 8.4%).

**Conclusions:**

This study shows that the number of medical specialists in Ecuador has increased significantly over the last two decades, although with inequalities in the distribution of specialists between provinces and regions. The results of this study provide background for the Ecuadorian health system when introducing Human Resources of Health (HRH) policies.

**Supplementary Information:**

The online version contains supplementary material available at 10.1186/s12913-022-08056-5.

## Introduction

The number and geographical distribution of physicians have a major impact on health provision [1]. There is extensive evidence showing that regions with a low density and poor distribution of medical professionals have negative health indicators, low coverage and limited accessibility [2, 3]. Although there is evidence that coverage with and distribution of physicians has improved over recent years in some Organization for Economic Co-operation and Development (OECD) countries, [3, 4] most Low and Middle-Income Countries (LMICs) still face workforce inequalities because of a concentration of physicians in urban areas and scarcity of professionals in rural areas [5, 6].

The shortage or surplus of physicians has been a primary concern in health sectors workforce planning and remains a controversial issue in many countries [1]. There is no clear benchmark to decide what is an adecuate number of physicians in any country or region [7]. However, the most common approach to monitoring the Health Workforce (HW) has been the workforce-to-population ratio method [1]. This approach estimates the current HW density or supply per 1,000 or 10,000 population (e.g. physicians per 1,000 population), and it has been widely used for comparative analyses across countries and regions [8]. Over recent years, the benchmarks of 23 physicians, nurses and midwives per 10,000 population has been commonly used as a minimum threshold for comparisons between countries [8]. However, in 2016 the World Health Organization (WHO) recommended a new parameter of human resources for health of 44.5 professionals per 10,000 population (including physicians, nurses, and midwives) [9]. In the context of Latin America (LA), only eight countries met this threshold in 2016: the Bahamas, Barbados, Brazil, Cuba, Grenada, Mexico, Trinidad and Tobago, and Uruguay [9].

In Ecuador, the number of HW has increased steadily in the last two decades [10]. For example, over the period 2000 to 2017, the number of physicians increased from 0.8 to 2.2 per 1,000 population [10]. However, although the rate of physicians in Ecuador has almost tripled since 2000, this rate is lower than the average rate of OECD countries of 3.5 physicians per 1,000 population [4]. In the case of LA, data available for 2015 showed that the average rate for the region was 2.2 physicians per 1,000 population, slightly higher than the 1.8 physicians per 1,000 populations in Ecuador in the same year [11].

In the specific case of medical specialists, there are limited data on the number of specialities and specialists in Ecuador. Currently, the country does not have indicators for these, and there are no studies to permit comparisons with other countries in the region [7]. The number and distribution of specialists is a measure of workforce availability and accessibility and are equity indicators in health systems performance and efficiency [1]. For a LMIC country like Ecuador, these indicators are important in identifying differences in geographical distributions, supply-and-demand projections, and definition of policies in medical education. The present study aimed to examine the temporal and geographical distribution of medical specialists in Ecuador over the period 2000 to 2017 and asses the implications of these findings for medical education.

## Methods

### Study design and population

We conducted an ecological time-series study based on administrative yearbooks databases provided by The National Institute of Statistics and Census (INEC). The study included all medical specialists working in Ecuador's public and private health institutions from 2000 to 2017.

### Study area and setting

**Fig. 1:**
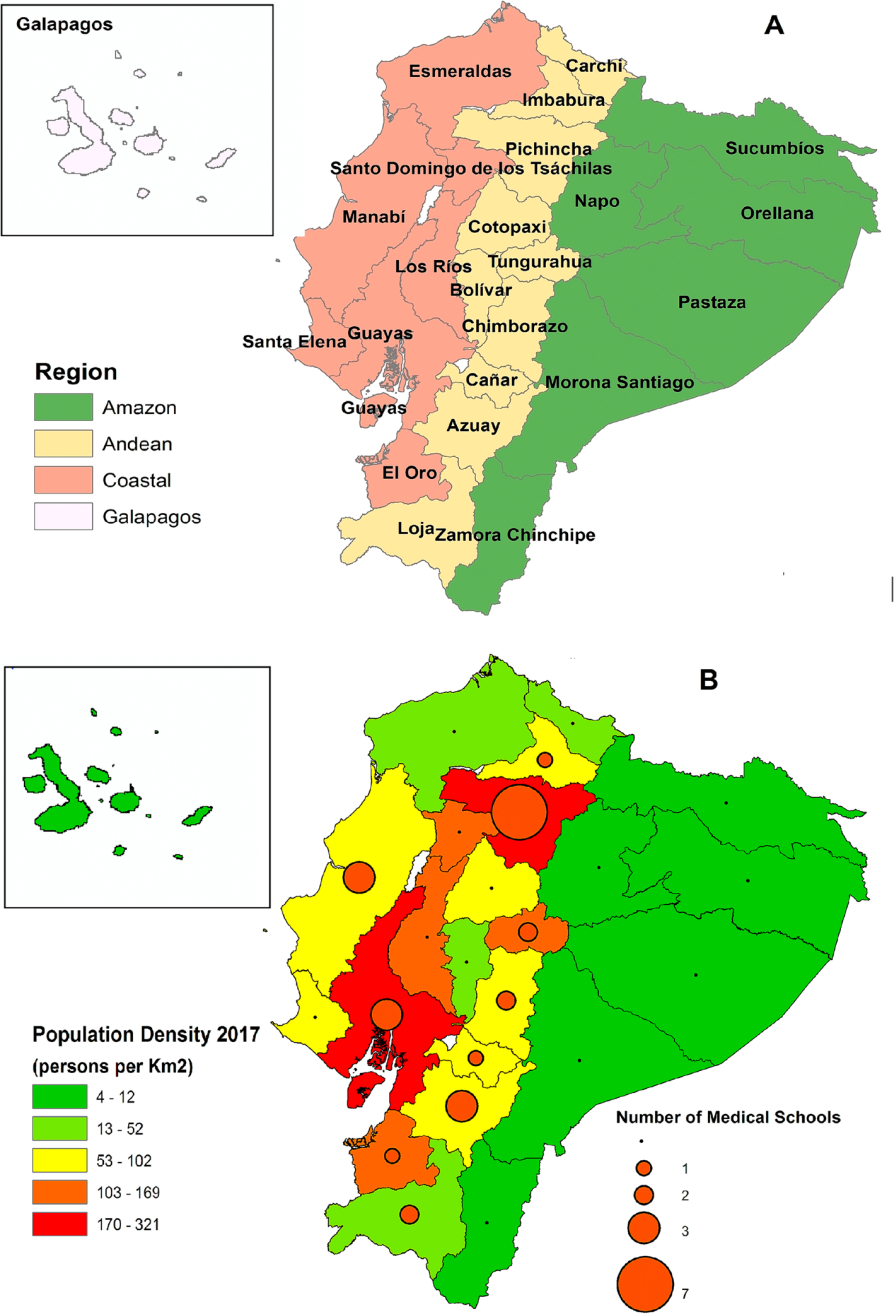
Map of Ecuador. A Administrative division of Ecuador by provinces and geographical region. B Population density by province and number of medical schools. Maps representing demographic characteristics of the country were built using ArcGIS version 10.2.2 (ESRI, California, USA)

Ecuador is an upper-middle-income country in South America with a per capita income of $6,080 in 2019 [12]. The country covers 283,560 km^2^ with a population density in 2017 of 70 per Km^2^. Ecuador has 24 provinces and four distinct geo-climatic regions: Andean, Amazon, Coastal, and the Galapagos Islands (Fig. 1). The country's total population is 17,510,643 based on projections for 2020, with a populational composition of 72% Mestizo (mixture of Spanish and indigenous), 7% Indigenous, 6% White, 7% Afro-Ecuadorian, and 8% others [13]. The three largest cities in the country are Quito with 2.7 million, Guayaquil with 2.3 million and Cuenca with 625,000 inhabitants (projections 2017) [13]. Petroleum and agriculture are the principal sources of income, with oil accounting for 40% of the country's exports [14]. The poverty rate in Ecuador in 2019 was 25% [12].

Ecuador has a fragmented health system that includes institutions funded by the government, social security, and private sectors [15]. Public institutions offer health care services to the entire population and are divided into four levels of care [16]. Social security institutions offer health services only through affiliated employees and their close relatives. The private sector consists of for-profit entities (hospitals, clinics, dispensaries, doctor's offices, pharmacies, and prepaid medicine companies) and is generally located in the larger cities [15]. According to INEC, there were 4,168 health institutions in the country in 2017, staffed by 37,293 doctors, mainly in the public sector [10]. Public and private health institutions report health and vital statistics data to the Ministry of Public Health and INEC.

### Data collection

We used data collected over a 18-year (2000 to 2017) period obtained from the Statistical Registry of Health Resources and Activities (RAS) [17]. RAS is an administrative yearbook for public and private health institutions (with and without hospitalisation) that collects relevant information on the health workforce, medical equipment, physical resources, and health surveillance [17]. For our analyses, we used data related to the health workforce: number of specialists, type of speciality, working days in hours and location.

**Fig. 2:**
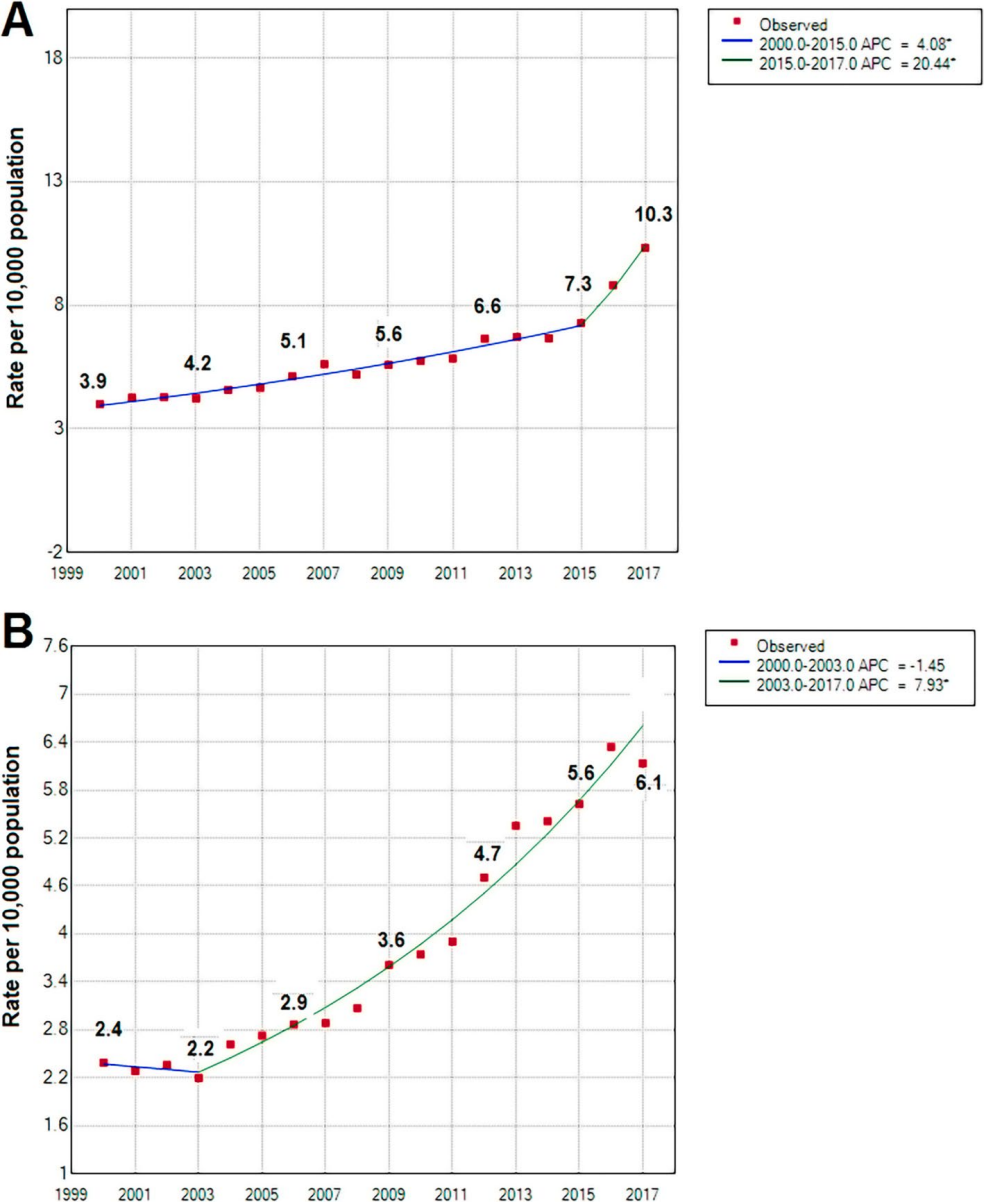
National trends in medical specialists (A) and graduate doctors (B), rates per 10,000 population over period 2000 – 2017. A National rate of medical specialists; B National rate of medical graduates. APC: Annual Percentage Change; * It indicates that the annual percentage change is significant (*p* < 0.05)

Medical specialists were defined as those professionals who have completed official medical studies programs, have completed specialised training in recognised area of medicine and have legal authorisation to exercise the profession in public and private health institutions [10]. Medical specialists were categorised into four groups: medical, surgical, diagnostic and other specialities. Additionally, we included in the analyses data on physicians in training (physicians undergoing medical specialisation, medical residents and physicians doing an obligatory year of rural medicine) [10]. The last group comprises doctors who have recently graduated from medical school. We defined two geographical indicators to evaluate the distribution of specialists across the country. 1) The first indicator corresponds to the country's administrative division in 24 provinces (See Fig. 1A), and 2) the second indicator considers the three biggest cities of Ecuador (Quito, Guayaquil, Cuenca) and the rest of the country as categories.

### Statistical analyses

The number of medical specialists was estimated based on their working hours in the health institutions to avoid double counting of professionals that work in more than one institution. The working day in Ecuador lasts 8 h. Therefore, those specialists who worked full time were counted as one unit (one specialist), those who worked 6 h a day were counted as 0.75 units; those who worked 4 h a day were counted as 0.50 units, and those who worked less than 4 h were counted as 0.25 units [10]. Finally, considering the speciality and year, ​​we added these values to estimate the total number of specialists.

For descriptive analyses, we calculated densities of medical specialists per 10,000 population at national, provincial, and area levels from 2000 to 2017. We also estimated rates by speciality and rates of physicians in training. Population estimations for the entire study period by country, province, and city were obtained from INEC. For trend analyses, Joinpoint regression models were used to evaluate rates of specialists and physicians in training [18]. This method identifies the year(s) when a trend change is produced by connecting several different line segments on a log scale at “joinpoints”. The analysis starts with zero joinpoints (i.e. a straight line) and then identifies points where a statistically significant change over time in the linear slope of the trend occurred, adding these points to the model. The Joinpoint method provides the Annual Percentage Change (APC) in rates between trend-change points and estimates the Average Annual Percentage Change (AAPC) over the whole study period. The APC is tested to determine if it differs from that expected under the null hypothesis (i.e. annual per cent change is 0%). In the final model, each joinpoint indicates a statistically significant change in trends (increase or decrease) and each of those trends is described by an APC. When there are no joinpoints (i.e. no changes in trend), APC is constant and equals AAPC [18].

We conducted two joinpoint models using the national rate of medical specialists and the rate of physicians in training. APC and AAPC were calculated with a 95% confidence interval (95% CI). P-value < 0.05 was considered statistically significant. Statistical analyses were done using Joinpoint software (Version 4.8.0.1) of the Surveillance Research Program of the US National Cancer Institute [19] and SPSS (Version 24). Maps representing demographic characteristics and medical distribution of the country were built using ArcGIS version 10.2.2 (ESRI, California, USA).

### Patient and public involvement

This analysis was based on anonymized secondary data from the Statistical Registry of Health Resources and Activities.

## Results

### Rates and trend analyses

Table 1 shows the number of specialists and specialities for 2000, 2006, 2012 and 2017. Our analyses identified 46 medical specialities registered in the public databases. The number of specialists in the country grew from 5005 to 17,313 between 2000 and 2017, representing a rate increase of 4 to 10.3 per 10,000 population (See Fig. 2A). In the same study period, physicians doing the rural year increased from 2.4 to 6.1 per 10,000 population (See Fig. 2B). The number and rates of physicians by speciality for the entire study period are described in Supplementary Tables ST1 and ST2. The Joinpoint analyses identified two temporal trends for the rate of medical specialists (See Fig. 2A). Between 2000 and 2015, specialists increased by 4.08% per year, and between 2015 and 2017, they increased 20.44% per year (both estimations with a *p*-value < 0.05). Likewise, the rate of physicians doing the rural year showed two temporal trends (See Fig. 2B). Between 2000 and 2003, the rate decreased by 1.45% per year, and between 2003 and 2017, the rate increased by 7.9% per year (*p* < 0.05).

**Table 1:** Estimated number of medical specialists for 2000, 2006, 2012 and 2017

	**Specialities**	**2000**	**2006**	**2012**	**2017**
**Medical group**	Internal Medicine	NA	331	655	886
	Cardiology	188	338	382	504
	Neurology	159	207	222	220
	Psychiatry	153	157	171	247
	Haematology	42	64	75	102
	Intensive Care Medicine	107	165	253	439
	Nephrology	53	75	108	248
	Pulmonology	70	115	123	140
	Gastroenterology	141	191	251	341
	Geriatric Medicine	12	38	42	75
	Medical Oncology	71	101	124	173
	Dermatology	NA	149	204	298
	Infectiology	NA	45	41	66
	Endocrinology	NA	70	98	166
	Allergology	NA	28	33	47
	Diabetology	NA	32	56	36
	Paediatrics	764	882	1311	1980
	Neonatology	107	134	222	250
	Obstetrics & Gynaecology	872	1105	1354	2076
	Family Medicine	NA	28	178	1454
	Nutrition	NA	NA	NA	54
	Rheumatology	NA	NA	NA	67
	Emergency Medicine	NA	NA	NA	580
	Critical Care	NA	NA	NA	15
	Sport and Exercise Medicine	NA	NA	NA	9
	Occupational Medicine	NA	NA	NA	239
	Physiatry	NA	NA	NA	219
**Surgical group**	Surgery-General	793	919	1090	1230
	Plastic Surgery	106	193	218	264
	Anaesthesiology	521	675	1012	1410
	Traumatology	361	444	580	604
	Ophthalmology/Otorhinolaryngology	332	414	458	597
	Urology	155	215	261	338
	Proctology	NA	NA	NA	38
	Vascular Surgery	NA	NA	NA	162
	Neurosurgery	NA	NA	NA	170
	Paediatric Surgery	NA	NA	NA	136
	Thoracic Surgery	NA	NA	NA	136
**Diagnostic group**	Pathology	NA	NA	286	312
	Radiology-Diagnostic	NA	NA	398	455
	Imageology	NA	NA	NA	398
	Nuclear Medicine	NA	NA	NA	12
**Other specialities**	Immunology	NA	NA	NA	6
	Medical Genetics	NA	NA	NA	27
	Epidemiology	NA	18	64	79
	Public Health	NA	24	41	15
**Medical specialist**	Total	5006	7157	10,308	17,313
**General Practitioners**	Total	3236	3132	6030	8910
**Medical Interns**	Total	NA	NA	776	1531
**Medical residents**	Total	2083	2303	4048	4909
**Recent Graduates**	Total	908	1699	2479	4149

*NA* Not available

### Geographical distributions of medical specialists

**Fig. 3:**
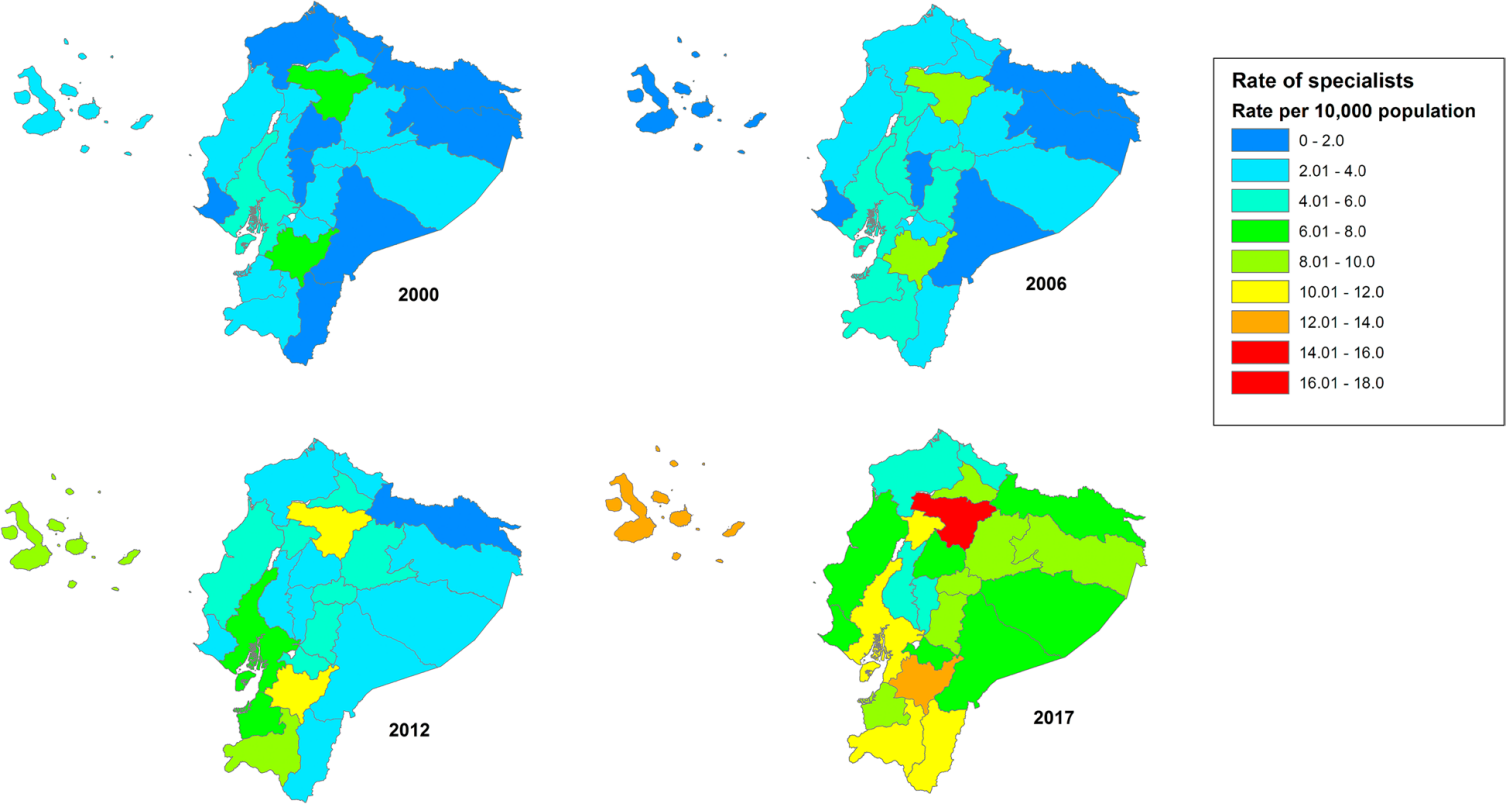
Rates of medical specialists per 10,000 inhabitants by province for 2000, 2006, 2012 and 2017. Maps representing medical distribution in the country were built using ArcGIS version 10.2.2 (ESRI, California, USA)

Table 2 shows the percent distribution of medical specialists in the three main cities of Ecuador compared to the rest of the country for each year of the study period. Over the entire study period, more than 50% of the medical specialists nationally were concentrated in the three largest cities of Quito, Guayaquil, and Cuenca. The number of medical specialists in other cities and rural areas of the country (Rest of the country category) increased from 1864 (37.2%) in 2000 to 7979 (46.1%) in 2017. Figure 3 shows the geographical distributions of medical specialists by provinces for 2000, 2006, 2012 and 2017. The figure shows that, for the entire study period, the provinces of Pichincha and Azuay had a higher rate of specialists compared to other provinces. In 2017, the provinces with the low numbers of specialists were Esmeraldas,

**Table 2:** Percentage distribution of medical specialists in four geographical areas of Ecuador, period 2000 – 2017

	**Cuenca** **n (%)**	**Guayaquil** **n (%)**	**Quito** **n (%)**	**Rest of the country** **n (%)**
2000	456 (9.1%)	1418 (28.3%)	1269 (25.3%)	1864 (37.2%)
2001	495 (9.1%)	1509 (27.7%)	1363 (25%)	2087 (38.3%)
2002	515 (9.2%)	1522 (27.2%)	1324 (23.6%)	2239 (40%)
2003	519 (9.2%)	1385 (24.6%)	1409 (25.1%)	2306 (41%)
2004	563 (9.1%)	1615 (26.1%)	1456 (23.5%)	2550 (41.2%)
2005	558 (8.7%)	1651 (25.8%)	1549 (24.2%)	2635 (41.2%)
2006	659 (9.2%)	1755 (24.5%)	1882 (26.3%)	2862 (40%)
2007	688 (8.6%)	1997 (25%)	1925 (24.1%)	3377 (42.3%)
2008	656 (8.7%)	1683 (22.4%)	1813 (24.2%)	3351 (44.7%)
2009	588 (7.2%)	2008 (24.4%)	1899 (23.1%)	3728 (45.3%)
2010	679 (7.9%)	2076 (24.1%)	1924 (22.3%)	3938 (45.7%)
2011	658 (7.4%)	2385 (26.7%)	2201 (24.7%)	3678 (41.2%)
2012	766 (7.4%)	2731 (26.5%)	2510 (24.3%)	4301 (41.7%)
2013	773 (7.3%)	2587 (24.4%)	2754 (26%)	4478 (42.3%)
2014	618 (5.8%)	2549 (23.9%)	2551 (23.9%)	4953 (46.4%)
2015	794 (6.7%)	2618 (22.1%)	3056 (25.8%)	5374 (45.4%)
2016	839 (5.8%)	3275 (22.5%)	3816 (26.2%)	6641 (45.6%)
2017	1005 (5.8%)	3967 (22.9%)	4363 (25.2%)	7979 (46.1%)

Carchi, Los Ríos, and Bolívar, each with less than six specialists per 10,0000 population. The figure also shows that the region with fewest specialists was the Amazon region.

### Percentage of professionals by medical specialisation

**Fig. 4:**
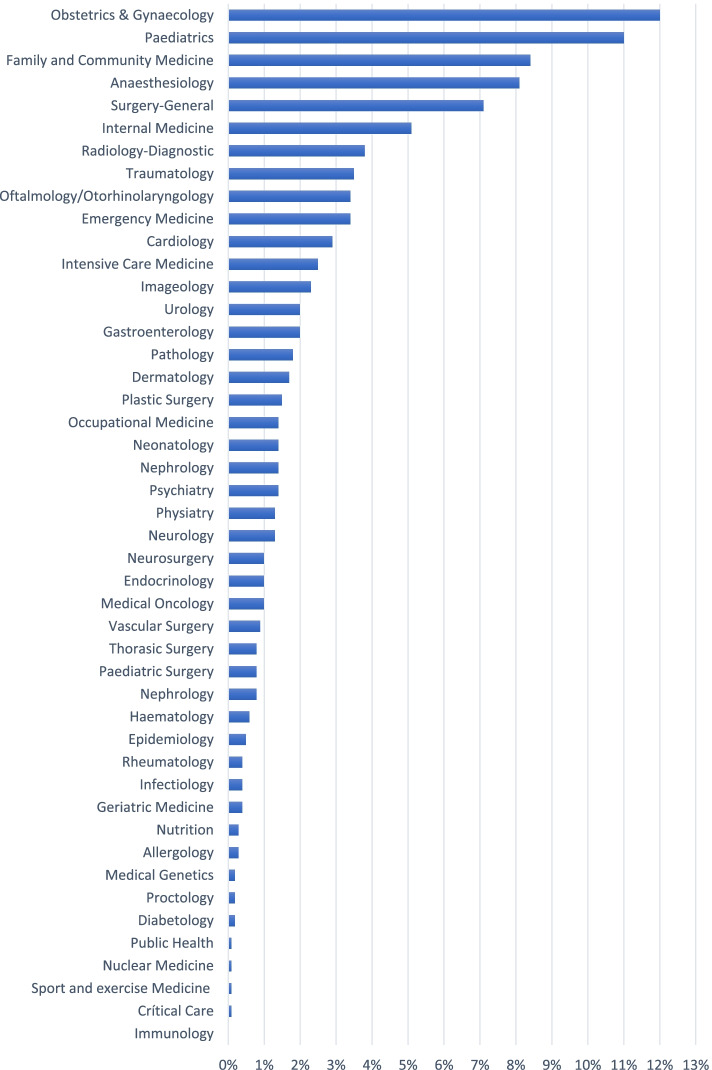
Percentage distribution of medical specialists in 2017

Figure 4 shows the percentage of physicians by specialisation in 2017. The specialities with the highest representation were gynaecology (12%), paediatrics (11%), family and community health (8.4%), general surgery (7.1%) and anaesthesiology (8.1%). The specialities with the lowest number of professionals were immunology, critical care, sport and exercise medicine, nuclear medicine, public health and diabetology. Specialities such as family and community medicine and epidemiology have significantly increased the number of professionals over the last few years.

## Discussion

In the present study, we conducted an ecological time-series analysis between 2000 and 2017 to describe the geographical distribution and temporal trends for medical specialists in Ecuador. Using the population ratio method, we estimated rates of medical specialists by year, geographical area, and groups of specialities. Our results showed that the quantity and density of specialists grew continuous during the analysis period but with clear differences in health care professional allocation. Our study identified the presence of more than 45 medical specialities in the country by 2017, of which the so-called “traditional specialities” accounted for more than 30% of professionals. However, other specialities such as family and community medicine and epidemiology have significantly increased over the last few years.

In Ecuador, the total number of doctors and other health professionals has grown steadily over the last 20 years [11]. Several demographic, socio-economic and political factors may explain this increase. Firstly, population growth directly impacts the demand for health services. In the case of Ecuador, between 2000 and 2017, the population increased from 12,531,210 to 16,776,977 [13]. Likewise, between 2006–2017, the number of medical consultations increased from 14,372,251 to 66,899,675, representing an 365% increase [20]. Secondly, over the last two decades, a series of policies and reforms aimed at increasing the population's social and health security coverage has led to a greater demand for medical specialists in the country [21, 22]. Finally, coupled with social reforms, the country also experienced major investments in health infrastructure [20]. For example, between 2009 and 2015, 47 hospitals and 74 health centres were built or repaired [20].

**Fig. 5:**
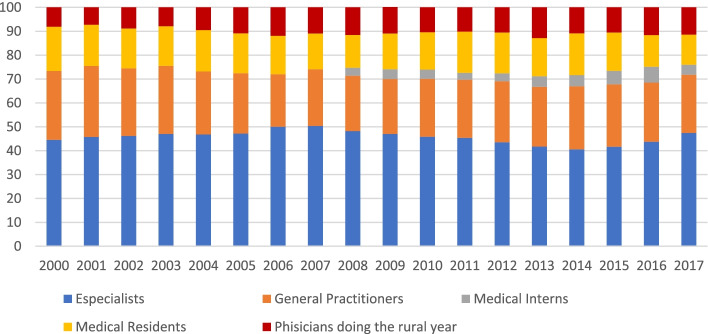
Percentage of specialists, general practitioners, medical intern, medical residents, and physician doing the rural year

In LA, several studies have addressed the number and the distribution of medical specialists using different methodologies [7, 23–28]. In the specific case of medical specialists in Ecuador, our results showed that the country experienced a growth rate of 158% between 2000 and 2017. This increase has caused concerns about a possible surplus of physicians in the country. However, comparing the Ecuadorian rate with rates in high-income countries and other countries in the region, we can see that the number of specialists in the country is relatively moderate. For example, in 2013, the average rate of medical specialists for countries in the European region was 21.3 per 10,000 population [29]. The Ecuadorian rate was 6.7 specialists per 10,000 population in the same year. In 2017, Australia, Denmark, Italy, Spain, and the United Kingdom had equivalent rates of 17.8, 17.9, 31, 25.3 and 20.5, respectively [11]. For the same year, the Ecuadorian rate was 10.3, a lower rate than those mentioned above. In the case of LA, the rate of medical specialists varies widely across countries. For example, the number of specialists in Ecuador in 2017 was higher than El Salvador, Honduras and Costa Rica, whose rates were 6, 1.4 and 6.7 specialists per 10,000 population, respectively [11]. However, countries such as Uruguay, Brazil, Mexico, and Chile (with rates of 25, 14.3, 15.2 and 12 specialists per 10,000 population, respectively) present relatively higher rates than Ecuador [11]. Our results also showed that (over the entire analysis period) medical specialists represented, on average, 46% of the total number of physicians (see Fig. 5). This percentage is lower than the OCED average, where medical specialists represent 65% of the total number of physicians [4]. The lower rate of specialists in Ecuador compared to some middle- and high-income countries, for now, do not suggest a surplus in the number of medical specialists.

In agreement with other studies, that observed an unequal geographical distribution in the number of specialists [2, 30–32], our study identified geographical differences in the number of specialists by region and province within Ecuador. Our results showed that more than 50% of the specialists were found in three largest cities (Quito, Guayaquil and Cuenca). Several studies have shown a greater concentration of medical professionals in large cities because these localities present more significant economic, social and professional incentives [23, 24, 32, 33]. However, despite an unequal distribution, the proportion of medical specialists in other cities and rural areas increased from 37 to 46% between 2000 and 2017. Part of this increase and redistribution of medical professionals in minor urban centres and rural areas could be related to health cooperation agreements between Ecuador and Cuba, where Cuban physicians were placed in the least densely populated areas of the country [34]. Another factor associated with the concentration of specialists in urban centres, although to a lesser extent, is the use of special equipment and materials generally found in hospitals in large cities. For example, radiologists' work requires expensive medical equipment such as computer tomography, magnetic resonance imaging, and positron emission tomography scanners [3].

Likewise, the geographical analysis by province showed marked inequalities throughout Ecuador (see Fig. 3). The provinces Pichincha, Azuay, Guayas and Loja had a higher concentration of specialists over the entire study period. In contrast, provinces as Esmeraldas, Carchi, Los Ríos and Bolívar showed a relative scarcity of medical specialists. The low number of medical specialists in these areas could be linked to social problems such as poverty and violence, especially in Esmeraldas and Carchi, which face drug trafficking and guerrilla problems due to their proximity to the Colombian border.

Although this study did not evaluate the structure of medical residencies and specialised training in Ecuador, it provides a broad frame of reference to identify specific policies for training specialists through the description of specialities and the number of specialists. According to our data for 2017, there were more than 45 specialities and subspecialties registered in the RAS database. However, the so-called “basic specialities” such as gynaecology, paediatrics and family and community medicine together represented almost 35% of total medical specialists. In contrast, other specialities such as geriatrics, rheumatology, or epidemiology did not exceed 1%. Such imbalances suggest that specialised training have not undergone similar changes in line with population growth and demand [7]. For example, increasing ageing of the population should generated a greater demand for medical care for the elderly, however, the number of specialists in geriatrics did not alter significantly over the analysis period. These disparities between the number of specific specialists and the epidemiologic and demographic characteristics of the population present a challenge for medical schools and health agencies responsible for the policy of HRH.

The amount of specialists in a country depends on the number of graduates from medical schools and the number of physicians practising medicine in the same country. In Ecuador, the number of physicians who graduated from medical schools (physicians doing the rural year) increased from 908 to 4609 between 2000 and 2017, increasing by 121%. The constant growth in medical schools plays a vital role in understanding the steady increase in medical specialists in the country. For example, over the past 30 years, medical schools have increased from 7 to 25 schools (with a national rate of 1.4 schools per 1,000,000 population) [35]. In the country, the training programs for medical specialists are offered by medical schools accredited by the government [36]. In 2014, the government implemented a standardised university accreditation system for the 22 medical schools. Currently, the country has 25 medical schools and 33 accredited medical specialist programs [35]. A standard habilitation test was implemented in 2014 to measure theoretical knowledge for medical graduates wanting to practice medicine in Ecuador. However, the admission system for specialisation programs is based on non-standardised tests. The graduation criteria from medical specialisations are tests or research papers. Most of the specialist training programs are self-financed. The Ministry of Public Health and private hospitals finance specific programs with repayment periods by graduates in the same institution. There is no standard habilitation test for medical specialists.

The present study has several limitations. 1) The study is based on secondary source, so information biases, specifically over-registration, must be considered. We minimised the risk of over-registration by counting the working hours of each professional and not the presence of the professionals by health institutions. Additionally, the data for this analysis comes from a single data source devised to collect information especially for health professionals, contrary to other studies that have used several sources of information to estimate the number of specialists [30]. 2) The RAS database does not provide detailed information at the individual level, such as nationality, age, sex, ethnicity and professional qualifications of health workers. The presence of demographic variables in the analysis would enrich the understanding of the differences in specialists' spatial and temporal distribution. Finally, the workforce-to-population ratio method does not consider variables such as disease burden, health care models, organisational efficiency, health policies, regulations and standards, technological capacity, among others. These variables profoundly modify the performance of medical specialists in health outcomes. Further studies of supply and demand for medical professionals are needed to overcome this limitation.

In conclusion, a country needs to know whether there is a shortage, surplus or inequitable geographical distribution in the medical workforce. However, there is no minimum number recommended by the WHO in the specific case of medical specialists [8]. The number and type of specialists respond to several factors such as the epidemiological profile of the population under study, type of health system, the demographic composition of the population, patient demand, advances in technology, medical knowledge and planning in medical residencies [25–28, 37]. So, what is the correct number of doctors? This question has several answers. It will fundamentally depend on the health system's vision and service delivery model. It is important to recognise that the health model itself conditions the need for professionals. Conversely, the availability of professionals can condition the provision of health care and its structuring [26]. Studies of supply and demand for medical professionals could provide the answers to the question of how many medical specialists a country needs. However, this methodology requires a detailed and rigorous analysis of numerous factors as the number of new medical students in the faculties, number and distribution of resident places, defined retirement policies, migration of professionals, strategies for territorial distribution of resources or policies on working conditions and professional motivation, among others [23].

## Conclusions

This study showed that medical specialists have increased significantly in Ecuador over the past two decades. However, specialists are mainly concentrated in large cities where healthcare facilities are more numerous and better resourced and where living conditions are superior. Rural areas and small urban settlements are underserved areas needing more specialised medical services. Although the present study has generated information to evaluate the distribution of the health workforce in the country, future studies must be conducted to examine the implications of the increase and distribution of specialists in the health system, health care policies, human resources of health, and the behaviour of the labour and education markets.

## Supplementary Information

**Additional file 1:** **SupplementaryTable ST1. **Estimated number of medical specialists and doctors in training,period 2000 - 2017. **Supplementary Table ST2. **Rates of medical specialists per 100,000 population, period 2000 – 2017. 

## Data Availability

Data on the number and rates of medical especialists are totally presented in the manuscript and the supplementary tables. Information about health national databases can be find in: https://www.ecuadorencifras.gob.ec/actividades-y-recursos-de-salud/.

## Abbreviations

LA: Latin America
INEC: National Institute of Statistical and Census of Ecuador
WHO: World Health Organization
APC: Annual Percentage Change
AAPC: Average Annual Percentage Change
PC: Percentual Change
OECD: Organization for Economic Co-operation and Development
HW: Health Workforce

## Declarations

### Concent Publications

Not applicable.F
