# Supplementary material for: Medical specialist distributions in Ecuador: a geographical and temporal analysis of data from 2000 to 2017
Source: BMC Health Serv Res. 2022 May 19;22:671. doi: 10.1186/s12913-022-08056-5 (PMC9118719; doi:10.1186/s12913-022-08056-5)
Supplement: Supplementary file 1 [file 12913_2022_8056_MOESM1_ESM.odt]

SUPLEMENTARY TABLES 

Supplementary Table ST1. Estimated number of medical specialists and doctors in training, period 2000 - 2017.

|  | Specialities | 2000 | 2001 | 2002 | 2003 | 2004 | 2005 | 2006 | 2007 | 2008 | 2009 | 2010 | 2011 | 2012 | 2013 | 2014 | 2015 | 2016 | 2017 |
| --- | --- | --- | --- | --- | --- | --- | --- | --- | --- | --- | --- | --- | --- | --- | --- | --- | --- | --- | --- |
| Medical group | Internal Medicine | - | - | - | - | - | - | 331 | 399 | 364 | 491 | 512 | 548 | 655 | 744 | 753 | 792 | 887 | 886 |
| Cardiology | 188 | 212 | 273 | 299 | 344 | 347 | 338 | 377 | 338 | 344 | 336 | 339 | 382 | 402 | 373 | 408 | 479 | 504 |
| Neurology | 159 | 110 | 171 | 172 | 159 | 149 | 207 | 221 | 188 | 198 | 187 | 192 | 222 | 233 | 199 | 215 | 232 | 220 |
| Psychiatry | 153 | 153 | 149 | 143 | 158 | 166 | 157 | 188 | 143 | 146 | 160 | 155 | 171 | 170 | 203 | 214 | 234 | 247 |
| Haematology | 42 | 48 | 55 | 53 | 58 | 61 | 64 | 69 | 59 | 59 | 76 | 65 | 75 | 81 | 73 | 82 | 96 | 102 |
| Intensive Care Medicine | 107 | 144 | 130 | 157 | 151 | 163 | 165 | 179 | 177 | 220 | 205 | 212 | 253 | 283 | 262 | 334 | 396 | 439 |
| Nephrology | 53 | 63 | 66 | 79 | 71 | 75 | 75 | 80 | 63 | 74 | 86 | 88 | 108 | 115 | 122 | 148 | 218 | 248 |
| Pulmonology | 70 | 75 | 87 | 85 | 91 | 96 | 115 | 116 | 98 | 107 | 111 | 114 | 123 | 127 | 120 | 121 | 129 | 140 |
| Gastroenterology | 141 | 151 | 162 | 180 | 183 | 184 | 191 | 206 | 191 | 208 | 216 | 229 | 251 | 279 | 271 | 293 | 315 | 341 |
| Geriatric Medicine | 12 | 15 | 16 | 17 | 23 | 20 | 38 | 31 | 32 | 34 | 37 | 31 | 42 | 56 | 61 | 64 | 70 | 75 |
| Medical Oncology | 71 | 77 | 78 | 79 | 83 | 116 | 101 | 102 | 100 | 105 | 95 | 106 | 124 | 108 | 109 | 141 | 146 | 173 |
| Dermatology | - | - | - | - | - | - | 149 | 171 | 143 | 152 | 166 | 164 | 204 | 221 | 214 | 220 | 442 | 298 |
| Infectiology | - | - | - | - | - | - | 45 | 35 | 34 | 27 | 37 | 39 | 41 | 38 | 44 | 49 | 58 | 66 |
| Endocrinology | - | - | - | - | - | - | 70 | 79 | 72 | 76 | 77 | 79 | 98 | 97 | 111 | 118 | 148 | 166 |
| Allergology | - | - | - | - | - | - | 28 | 33 | 37 | 31 | 38 | 28 | 33 | 29 | 34 | 37 | 39 | 47 |
| Diabetology | - | - | - | - | - | - | 32 | 39 | 50 | 41 | 53 | 53 | 56 | 48 | 38 | 45 | 41 | 36 |
| Paediatrics | 764 | 791 | 800 | 845 | 946 | 933 | 882 | 979 | 879 | 950 | 1030 | 1099 | 1311 | 1317 | 1356 | 1419 | 1848 | 1980 |
| Neonatology | 107 | 119 | 132 | 121 | 145 | 150 | 134 | 191 | 129 | 167 | 161 | 179 | 222 | 269 | 182 | 197 | 244 | 250 |
| Obstetrics & Gynaecology | 872 | 964 | 964 | 941 | 1058 | 1075 | 1105 | 1199 | 1006 | 1092 | 1115 | 1215 | 1354 | 1387 | 1508 | 1711 | 1916 | 2076 |
| Community & Family Medicine | - | - | - | - | - | - | 28 | 56 | 63 | 88 | 127 | 147 | 178 | 169 | 341 | 683 | 901 | 1454 |
| Nutrition | - | - | - | - | - | - | - | - | - | - | - | - | - | - | - | - | 30 | 54 |
| Rheumatology | - | - | - | - | - | - | - | - | - | - | - | - | - | - | - | - | 58 | 67 |
| Emergency Medicine | - | - | - | - | - | - | - | - | - | - | - | - | - | - | - | - | - | 580 |
| Critical Care | - | - | - | - | - | - | - | - | - | - | - | - | - | - | - | - | - | 15 |
| Sport and Exercise Medicine | - | - | - | - | - | - | - | - | - | - | - | - | - | - | - | - | - | 9 |
| Occupational Medicine | - | - | - | - | - | - | - | - | - | - | - | - | - | - | - | - | - | 239 |
| Physiatry | - | - | - | - | - | - | - | - | - | - | - | - | - | - | - | - | 167 | 219 |
| TOTAL | 2737 | 2921 | 3083 | 3170 | 3471 | 3533 | 4256 | 4749 | 4165 | 4609 | 4822 | 5081 | 5900 | 6173 | 6375 | 7290 | 9092 | 10929 |

Supplementary Table 1 ST1. Continuation 

|  | Specialities | 2000 | 2001 | 2002 | 2003 | 2004 | 2005 | 2006 | 2007 | 2008 | 2009 | 2010 | 2011 | 2012 | 2013 | 2014 | 2015 | 2016 | 2017 |
| --- | --- | --- | --- | --- | --- | --- | --- | --- | --- | --- | --- | --- | --- | --- | --- | --- | --- | --- | --- |
| Surgical group | Surgery-General | 793 | 897 | 827 | 812 | 916 | 986 | 919 | 1026 | 921 | 951 | 970 | 993 | 1090 | 1115 | 1058 | 1036 | 1138 | 1230 |
| Plastic Surgery | 106 | 130 | 130 | 127 | 151 | 146 | 193 | 211 | 190 | 188 | 195 | 185 | 218 | 208 | 185 | 188 | 230 | 264 |
| Anaesthesiology | 521 | 563 | 565 | 570 | 609 | 647 | 675 | 773 | 749 | 827 | 868 | 905 | 1012 | 1063 | 1041 | 1132 | 1305 | 1410 |
| Traumatology | 361 | 406 | 410 | 394 | 458 | 451 | 444 | 475 | 448 | 480 | 520 | 504 | 580 | 569 | 585 | 623 | 725 | 604 |
| Ophthalmology/Otorhinolaryngology | 332 | 345 | 344 | 333 | 354 | 388 | 414 | 435 | 387 | 391 | 415 | 403 | 458 | 462 | 443 | 513 | 613 | 597 |
| Urology | 155 | 191 | 241 | 215 | 226 | 242 | 215 | 241 | 214 | 237 | 233 | 233 | 261 | 265 | 257 | 268 | 320 | 338 |
| Proctology | - | - | - | - | - | - | - | - | - | - | - | - | - | - | - | - | - | 38 |
| Vascular Surgery | - | - | - | - | - | - | - | - | - | - | - | - | - | - | - | - | 124 | 162 |
| Neurosurgery | - | - | - | - | - | - | - | - | - | - | - | - | - | - | - | - | 146 | 170 |
| Paediatric Surgery | - | - | - | - | - | - | - | - | - | - | - | - | - | - | - | - | - | 136 |
| Thoracic Surgery | - | - | - | - | - | - | - | - | - | - | - | - | - | - | - | - | 40 | 136 |
| TOTAL | 2268 | 2533 | 2517 | 2450 | 2714 | 2860 | 2860 | 3162 | 2909 | 3074 | 3202 | 3223 | 3619 | 3681 | 3569 | 3760 | 4640 | 5082 |
| Diagnostic group | Pathology | - | - | - | - | - | - | - | - | 159 | 184 | 212 | 228 | 286 | 260 | 268 | 275 | 281 | 312 |
| Radiology-Diagnostic | - | - | - | - | - | - | - | - | 199 | 267 | 294 | 310 | 398 | 351 | 383 | 437 | 478 | 455 |
| Imageology | - | - | - | - | - | - | - | - | - | - | - | - | - | - | - | - | - | 398 |
| Nuclear Medicine | - | - | - | - | - | - | - | - | - | - | - | - | - | - | - | - | - | 12 |
| TOTAL | - | - | - | - | - | - | - | - | 358 | 451 | 506 | 538 | 684 | 611 | 650 | 713 | 759 | 1176 |
| Other specialities | Immunology | - | - | - | - | - | - | - | - | - | - | - | - | - | - | - | - | - | 6 |
| Medical Genetics | - | - | - | - | - | - | - | - | - | - | - | - | - | - | - | - | - | 27 |
| Epidemiology | - | - | - | - | - | - | 18 | 26 | 26 | 45 | 46 | 50 | 64 | 88 | 50 | 51 | 56 | 79 |
| Public Health | - | - | - | - | - | - | 24 | 50 | 44 | 43 | 41 | 29 | 41 | 40 | 25 | 29 | 24 | 15 |
| TOTAL | - | - | - | - | - | - | 41 | 76 | 71 | 88 | 87 | 79 | 106 | 128 | 76 | 80 | 80 | 126 |
| Physician in training | Training in specialisation | - | - | - | - | - | - | - | - | 516 | 729 | 732 | 574 | 776 | 1125 | 1226 | 1620 | 2213 | 1531 |
| Medical Residents | 2083 | 2059 | 2027 | 1980 | 2292 | 2268 | 2303 | 2371 | 2134 | 2620 | 2937 | 3389 | 4048 | 4058 | 4591 | 4556 | 4411 | 4609 |
| Year of rural medicine | 908 | 865 | 1066 | 945 | 1253 | 1472 | 1699 | 1729 | 1795 | 1976 | 1949 | 1995 | 2479 | 3260 | 2854 | 2977 | 3851 | 4149 |
| TOTAL | 2992 | 2924 | 3093 | 2925 | 3545 | 3740 | 4002 | 4099 | 4445 | 5324 | 5618 | 5958 | 7303 | 8443 | 8670 | 9154 | 10475 | 10289 |

Supplementary Table ST2. Rates of  medical specialists per 100,000  population, period 2000 – 2017.

| Grupo | Especialidades | 2000 | 2001 | 2002 | 2003 | 2004 | 2005 | 2006 | 2007 | 2008 | 2009 | 2010 | 2011 | 2012 | 2013 | 2014 | 2015 | 2016 | 2017 |
| --- | --- | --- | --- | --- | --- | --- | --- | --- | --- | --- | --- | --- | --- | --- | --- | --- | --- | --- | --- |
| Medical group | Internal Medicine |  |  |  |  |  |  | 2.37 | 2.8 | 2.51 | 3.33 | 3.41 | 3.59 | 4.22 | 4.72 | 4.7 | 4.87 | 5.36 | 5.28 |
| Cardiology | 1.5 | 1.66 | 2.08 | 2.25 | 2.54 | 2.53 | 2.42 | 2.65 | 2.34 | 2.33 | 2.24 | 2.22 | 2.46 | 2.55 | 2.33 | 2.5 | 2.9 | 3.0 |
| Neurology | 1.27 | 0.86 | 1.31 | 1.29 | 1.17 | 1.08 | 1.48 | 1.55 | 1.3 | 1.34 | 1.24 | 1.26 | 1.43 | 1.48 | 1.24 | 1.32 | 1.4 | 1.31 |
| Psychiatry | 1.22 | 1.2 | 1.14 | 1.07 | 1.17 | 1.21 | 1.12 | 1.32 | 0.99 | 0.99 | 1.07 | 1.02 | 1.1 | 1.08 | 1.27 | 1.31 | 1.42 | 1.47 |
| Haematology | 0.33 | 0.37 | 0.42 | 0.4 | 0.43 | 0.44 | 0.46 | 0.49 | 0.41 | 0.4 | 0.5 | 0.42 | 0.48 | 0.52 | 0.46 | 0.51 | 0.58 | 0.61 |
| Intensive Care Medicine | 0.85 | 1.12 | 0.99 | 1.18 | 1.11 | 1.19 | 1.18 | 1.26 | 1.22 | 1.49 | 1.36 | 1.39 | 1.63 | 1.79 | 1.63 | 2.05 | 2.4 | 2.61 |
| Nephrology | 0.42 | 0.49 | 0.51 | 0.59 | 0.53 | 0.55 | 0.54 | 0.56 | 0.44 | 0.5 | 0.57 | 0.57 | 0.69 | 0.73 | 0.76 | 0.91 | 1.32 | 1.48 |
| Pulmonology | 0.56 | 0.58 | 0.66 | 0.64 | 0.67 | 0.7 | 0.82 | 0.81 | 0.68 | 0.72 | 0.74 | 0.74 | 0.79 | 0.81 | 0.75 | 0.74 | 0.78 | 0.83 |
| Gastroenterology | 1.12 | 1.18 | 1.24 | 1.35 | 1.35 | 1.34 | 1.37 | 1.45 | 1.32 | 1.41 | 1.44 | 1.50 | 1.62 | 1.77 | 1.69 | 1.80 | 1.91 | 2.03 |
| Geriatric Medicine | 0.1 | 0.12 | 0.12 | 0.12 | 0.17 | 0.15 | 0.27 | 0.22 | 0.22 | 0.23 | 0.24 | 0.21 | 0.27 | 0.36 | 0.38 | 0.39 | 0.42 | 0.45 |
| Medical Oncology | 0.56 | 0.6 | 0.6 | 0.59 | 0.61 | 0.85 | 0.72 | 0.72 | 0.69 | 0.71 | 0.63 | 0.69 | 0.8 | 0.68 | 0.68 | 0.86 | 0.88 | 1.03 |
| Dermatology | - | - | - | - | - | - | 1.07 | 1.2 | 0.99 | 1.03 | 1.1 | 1.07 | 1.31 | 1.4 | 1.34 | 1.35 | 2.68 | 1.78 |
| Infectiology | - | - | - | - | - | - | 0.32 | 0.25 | 0.24 | 0.18 | 0.25 | 0.26 | 0.27 | 0.24 | 0.28 | 0.30 | 0.35 | 0.40 |
| Endocrinology | - | - | - | - | - | - | 0.50 | 0.55 | 0.50 | 0.52 | 0.51 | 0.52 | 0.63 | 0.61 | 0.69 | 0.72 | 0.89 | 0.99 |
| Allergology | - | - | - | - | - | - | 0.20 | 0.23 | 0.26 | 0.21 | 0.25 | 0.18 | 0.21 | 0.18 | 0.21 | 0.23 | 0.24 | 0.28 |
| Diabetology | - | - | - | - | - | - | 0.23 | 0.28 | 0.34 | 0.28 | 0.35 | 0.35 | 0.36 | 0.31 | 0.24 | 0.28 | 0.25 | 0.22 |
| Paediatrics | 6.10 | 6.17 | 6.11 | 6.34 | 6.98 | 6.80 | 6.32 | 6.89 | 6.07 | 6.44 | 6.86 | 7.20 | 8.45 | 8.35 | 8.46 | 8.72 | 11.2 | 11.80 |
| Neonatology | 0.85 | 0.93 | 1.01 | 0.91 | 1.07 | 1.09 | 0.96 | 1.34 | 0.89 | 1.14 | 1.08 | 1.17 | 1.43 | 1.71 | 1.14 | 1.21 | 1.47 | 1.49 |
| Obstetrics & Gynaecology | 6.96 | 7.52 | 7.36 | 7.06 | 7.81 | 7.83 | 7.91 | 8.39 | 6.95 | 7.41 | 7.43 | 7.96 | 8.72 | 8.79 | 9.41 | 10.5 | 11.6 | 12.37 |
| Community & Family Medicine | - | - | - | - | - | - | 0.20 | 0.39 | 0.43 | 0.60 | 0.84 | 0.96 | 1.14 | 1.07 | 2.13 | 4.20 | 5.45 | 8.67 |
| Nutrition | - | - | - | - | - | - | - | - | - | - | - | - | - | - | - | - | 0.18 | 0.32 |
| Rheumatology | - | - | - | - | - | - | - | - | - | - | - | - | - | - | - | - | 0.35 | 0.40 |
| Emergency Medicine | - | - | - | - | - | - | - | - | - | - | - | - | - | - | - | - | - | 3.46 |
| Critical Care | - | - | - | - | - | - | - | - | - | - | - | - | - | - | - | - | - | 0.09 |
| Sport and Exercise Medicine | - | - | - | - | - | - | - | - | - | - | - | - | - | - | - | - | - | 0.05 |
| Occupational Medicine | - | - | - | - | - | - | - | - | - | - | - | - | - | - | - | - | - | 1.43 |
| Physiatry | - | - | - | - | - | - | - | - | - | - | - | - | - | - | - | - | 1.01 | 1.30 |

Tabla Suplementaria TS2. Continuación

| Grupos | Especialidades | 2000 | 2001 | 2002 | 2003 | 2004 | 2005 | 2006 | 2007 | 2008 | 2009 | 2010 | 2011 | 2012 | 2013 | 2014 | 2015 | 2016 | 2017 |
| --- | --- | --- | --- | --- | --- | --- | --- | --- | --- | --- | --- | --- | --- | --- | --- | --- | --- | --- | --- |
| Surgical Group | Surgery-General | 6.33 | 7.00 | 6.32 | 6.10 | 6.76 | 7.18 | 6.58 | 7.22 | 6.36 | 6.45 | 6.46 | 6.51 | 7.02 | 7.07 | 6.60 | 6.36 | 6.89 | 7.33 |
| Plastic Surgery | 0.84 | 1.02 | 0.99 | 0.95 | 1.11 | 1.07 | 1.38 | 1.49 | 1.31 | 1.28 | 1.3 | 1.21 | 1.4 | 1.32 | 1.16 | 1.16 | 1.39 | 1.57 |
| Anaesthesiology | 4.16 | 4.40 | 4.31 | 4.28 | 4.49 | 4.71 | 4.84 | 5.44 | 5.17 | 5.61 | 5.78 | 5.93 | 6.52 | 6.74 | 6.49 | 6.95 | 7.89 | 8.4 |
| Traumatology | 2.88 | 3.17 | 3.13 | 2.96 | 3.38 | 3.28 | 3.18 | 3.34 | 3.10 | 3.26 | 3.47 | 3.30 | 3.74 | 3.61 | 3.65 | 3.83 | 4.39 | 3.6 |
| Ophthalmology/Otorhinolaryngolo | 2.65 | 2.70 | 2.63 | 2.50 | 2.62 | 2.83 | 2.96 | 3.06 | 2.68 | 2.65 | 2.76 | 2.64 | 2.95 | 2.93 | 2.76 | 3.15 | 3.71 | 3.56 |
| Urology | 1.24 | 1.49 | 1.84 | 1.61 | 1.67 | 1.77 | 1.54 | 1.7 | 1.48 | 1.61 | 1.55 | 1.53 | 1.68 | 1.68 | 1.6 | 1.65 | 1.94 | 2.01 |
| Proctology | - | - | - | - | - | - | - | - | - | - | - | - | - | - | - | - | - | 0.23 |
| Vascular Surgery | - | - | - | - | - | - | - | - | - | - | - | - | - | - | - | - | 0.75 | 0.96 |
| Neurosurgery | - | - | - | - | - | - | - | - | - | - | - | - | - | - | - | - | 0.88 | 1.01 |
| Paediatric Surgery | - | - | - | - | - | - | - | - | - | - | - | - | - | - | - | - | - | 0.81 |
| Thoracic Surgery | - | - | - | - | - | - | - | - | - | - | - | - | - | - | - | - | 0.24 | 0.81 |
| Diagnostic group | Pathology | - | - | - | - | - | - | - | - | 1.10 | 1.25 | 1.41 | 1.49 | 1.84 | 1.65 | 1.67 | 1.69 | 1.70 | 1.86 |
| Radiology-Diagnostic | - | - | - | - | - | - | - | - | 1.38 | 1.81 | 1.96 | 2.03 | 2.57 | 2.22 | 2.39 | 2.69 | 2.89 | 2.71 |
| Imageology | - | - | - | - | - | - | - | - | - | - | - | - | - | - | - | - | - | 2.37 |
| Nuclear Medicine | - | - | - | - | - | - | - | - | - | - | - | - | - | - | - | - | - | 0.07 |
| Other specialities | Immunology | - | - | - | - | - | - | - | - | - | - | - | - | - | - | - | - | - | 0.04 |
| Medical Genetics | - | - | - | - | - | - | - | - | - | - | - | - | - | - | - | - | - | 0.16 |
| Epidemiology | - | - | - | - | - | - | 0.13 | 0.18 | 0.18 | 0.31 | 0.30 | 0.33 | 0.41 | 0.56 | 0.31 | 0.31 | 0.34 | 0.47 |
| Public Health | - | - | - | - | - | - | 0.17 | 0.35 | 0.31 | 0.29 | 0.27 | 0.19 | 0.27 | 0.25 | 0.16 | 0.18 | 0.15 | 0.09 |
